# Live Imaging Of *Drosophila melanogaster *Embryonic Hemocyte Migrations

**DOI:** 10.3791/1696

**Published:** 2010-02-12

**Authors:** Iwan R. Evans, Jennifer Zanet, Will Wood, Brian M. Stramer

**Affiliations:** Department of Biology and Biochemistry, University of Bath; Randall Division of Cell and Molecular Biophysics, King's College London

## Abstract

Many studies address cell migration using *in vitro *methods, whereas the physiologically relevant environment is that of the organism itself. Here we present a protocol for the mounting of *Drosophila melanogaster *embryos and subsequent live imaging of fluorescently labeled hemocytes, the embryonic macrophages of this organism. Using the Gal4-uas system^1^ we drive the expression of a variety of genetically encoded, fluorescently tagged markers in hemocytes to follow their developmental dispersal throughout the embryo. Following collection of embryos at the desired stage of development, the outer chorion is removed and the embryos are then mounted in halocarbon oil between a hydrophobic, gas-permeable membrane and a glass coverslip for live imaging. In addition to gross migratory parameters such as speed and directionality, higher resolution imaging coupled with the use of fluorescent reporters of F-actin and microtubules can provide more detailed information concerning the dynamics of these cytoskeletal components.

**Figure:**
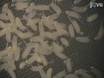


## Protocol

### Preparation

Obtain appropriate *Drosophila* lines containing a hemocyte-specific Gal4 driver (e.g. *srp-Gal4*^2^) and a genetically encoded fluorescent reporter under UAS control (e.g. *uas-GFP*). Flies homozygous for *srp-Gal4*,*uas-GMA*^3^ or *crq-Gal4,uas-GFP*^4, 5^ are particularly useful for imaging purposes (n.b. GMA is GFP fused to the actin-binding domain of moesin); see below for a discussion of the range of Gal4 drivers and uas constructs available (the Bloomington Stock Centre contains a wide variety).
Typically genetic crosses are carried out such that mutant alleles are balanced using fluorescent balancers *CTG* or *TTG*^6^, with Gal4 drivers and uas constructs carried on alternative homologous chromosomes. This makes it possible to select homozygous mutants on the basis of absence of *CTG*/*TTG*-associated GFP fluorescence (this is done at stage 2.11).
Amplify stocks and place flies in a laying cage with an apple juice agar plate^7^. The flies need at least two days to acclimatize to the laying cage before enough embryos begin to be laid. In general twenty flies of each sex should be sufficient to generate enough embryos for imaging but it should be noted that different lines have differing degrees of fertility. We use 55mm Petri dishes that fit into the bottom of a plastic beaker, punctured at its base to allow airflow. The exact means of embryo collection is unimportant, but the timings are critical in order to collect correctly staged embryos.
Collect embryos from an overnight apple juice agar plate maintained at 25°C or from a timed plate. For the latter we typically allow the flies to lay on a pre-warmed plate for 4 hours, before removing the plate and placing it at 18°C for 15-16 hours prior to mounting of embryos; this provides embryos from late stage 12 through to stage 15 of development. An overnight plate contains a greater diversity of stages but offers the advantage of higher levels of fluorescent reporter expression in hemocytes due to a longer period of time at 25°C as the Gal4-UAS system is temperature sensitive.

### Procedure

Dislodge embryos from the apple juice agar plate using a small amount of water and a soft-tipped paintbrush. Dislodged embryos can be seen easily with the naked eye.
Transfer embryos to a cell strainer (Fisher) or home-made basket^7^ by pouring water from the apple juice agar plate into the basket held over a beaker to collect waste water.
Repeat step 2.2 until you are satisfied you have enough embryos transferred from the apple juice agar plate.
Wash embryos in cell strainer/basket using water.
Place cell strainer/basket in the Petri dish lid of the apple juice agar plate and add enough neat bleach to suspend embryos in the cell strainer/basket.
Follow dechorionation of the embryos on a dissection microscope under brightfield: dechorionation is complete when the dorsal appendages have dissolved, which should occur within two minutes.
Remove cell strainer/basket containing embryos from the bleach and wash off residual bleach using water. All traces of bleach should be removed before proceeding to step 2.8. One trick to assess whether all the bleach has been removed is to blot off residual water on blue-colored laboratory tissues - if there is residual bleach the blue color will be bleached white/pink.
Blot off remaining water using laboratory tissue/mediwipes applied to the underside of the cell strainer/basket.
Place a droplet of water in a Petri dish lid. With a fine paintbrush, collect all the dechorionated embryos from the embryo basket and resuspend them in the droplet. Next dry the embryos by aspirating water using a micropipette or carefully absorbing it with a laboratory tissue/mediwipes.
Once the embryos have been dried, add a drop of voltalef oil to cover all the embryos. Put a second small drop of oil adjacent to the droplet containing the embryos. N.B. we have been unable to find a UK-based supplier of voltalef oil; halocarbon oil 700 (Sigma) may be used instead.
Under a fluorescent dissection microscope select appropriately staged embryos of the desired genotype using a pair of watchmakers forceps (number 5) from the oil droplet. These forceps should be bent inwards (Figure 1) in order to scoop up the embryos without puncturing their vitelline membrane. Transfer selected embryos to the second oil droplet. It is important that you are able to see fluorescent hemocytes on the dissecting microscope in order to be able to collect good images on the confocal microscope (Figure 2). We typically mount stage 13/14 embryos to image lateral migration of hemocytes on the ventral midline or stage 15 embryos to image the motility of hemocytes following dispersal over the embryo.
Stick two coverslips (18x18mm, thickness 1) to the underside of a Petriperm/Lumox dish (Sarstedt), using 2 small drops of voltalef oil, leaving approximately 1cm between them (Figure 3); these will be used to support a coverslip placed over the embryos, so as not to crush them. Petriperm dishes (50mm diameter) contain a hydrophobic, gas-permeable membrane. We find that the dishes become easier to use once they have been used several times (dishes can be wiped with 70% ethanol and reused).
Under brightfield on the dissection microscope, pick up selected embryos one by one with the bent forceps and line them ventral side up and parallel to the edge of the coverslips (Figure 3). It is possible to align up to 15 embryos in this way, depending on your dexterity and your patience. It is important to manipulate the embryos gently as both the embryos and Petriperm dish membrane are fragile and can be easily ruptured.
Once the embryos are aligned add a small drop of oil and let it spread to form a homogenous layer between the two coverslips. After the oil has spread (this may take a few minutes) check that the embryos are still ventral side up. If the embryos have rolled slightly, reposition them again with the forceps.
Finally, using tweezers (number 3) place a coverslip (18x18mm, thickness 1) over the embryos, resting upon the two previously adhered coverslips. Glue this coverslip to the coverslip supports using nail polish (Figure 3).
Take the Petriperm dish with mounted embryos to the confocal or wide-field microscope and mount the Petriperm dish on the stage using an appropriate adapter. Either an upright or inverted microscope may be used, with the objective lens focusing through the coverslip (as opposed to through the membrane).

### Representative Result:

This protocol describes how to mount Drosophila embryos for live imaging of hemocytes on the ventral side of the embryo. If done correctly it will be easy to generate either stills or movies of hemocytes. The major determinant is the microscope used to image the hemocytes (in particular the objective lens), but the nature of the images acquired will also depend upon the stage of development, the temperature the embryos were raised at and the Gal4 and uas lines used.

Higher levels of fluorescent protein expression will enable hemocytes to be imaged with greater ease, therefore it is important to be able to see hemocytes when the embryos are at stage 2.11 of the protocol (Figure 2 contains examples of clear hemocytes within embryos, taken with a camera fitted to a dissection microscope). Therefore increased numbers of Gal4 and uas constructs enable a greater signal-to-noise ratio. Furthermore this reduces the need for high laser intensities or increased exposure times when imaging, which in turn enables hemocyte behavior to be followed for longer periods of time.

Very high levels of GFP expression will show up fine details of hemocyte morphology, particularly the thin sheet-like lamellae that surround the circular cell body (Figure 4A-B). Circular regions excluding GFP represent phagosomes (Figure 4A-C). Finger-like filopodia can also be seen emerging from the lamellae (Figure 4B). Two Gal4 drivers remain sufficient to see these processes (Figure 4C), particularly if one or more is *srp-Gal4* (see discussion), however slower scanning speeds or greater laser power on the confocal microscope may be required. As expression levels decrease it becomes more difficult to image the protrusions of hemocytes; nonetheless it is still possible to track the migration of hemocytes under these conditions as the cell body remains obvious even when protrusions are less clear (Figure 4D).

At earlier stages of development (up to stage 13) hemocytes migrate in close contact to one another and it is often hard to distinguish individual cells. By the end of stage 13 hemocytes have formed a single line down the ventral midline (Figure 5A), then, becoming more motile, migrate laterally to the edges of the ventral nerve cord (Figure 5B). The actin cytoskeleton within the dynamic protrusions of hemocytes can be directly observed through expression of GMA (Figure 5C) or cherry-moesin.

Mounting the embryos in this way allows gas exchange and prevents dehydration and embryos remain viable following imaging. If the embryo is damaged during mounting it is generally obvious as the embryo content will leak through its vitelline membrane. If an embryo does start to dehydrate then this can often been seen by deformations in the vitelline membrane. Occasionally an embryo will roll during the course of a timelapse movie, however this only tends to be problematic for longer timescale movies. Lastly, mounting several embryos at once gives the experimentalist the best chance of obtaining an embryo in the perfect orientation for their experiment.


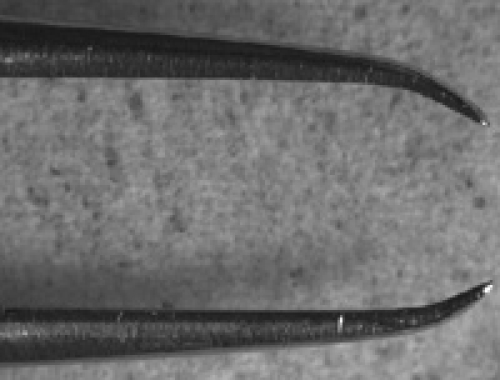
 **Figure 1. Forceps for manipulation of dechorionated embryos**.The tips of watchmakers forceps (size number 5) should be bent inwards in order to fashion a tool to scoop up embryos as shown here. The outer surface of the bent region is also useful to manipulate embryos when positioning on the Petriperm membrane as they possess no sharp edges that could puncture the embryo.


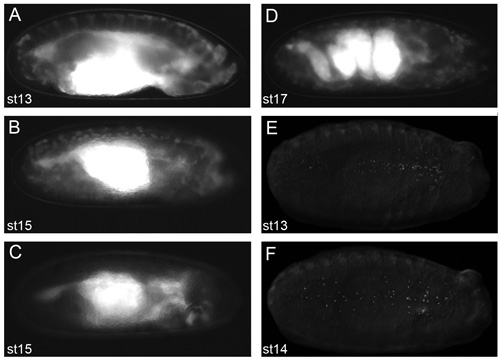
 **Figure 2. Representative images of embryos that will yield good live imaging results**.Images of dechorionated embryos in voltalef oil (at stage 2.11 of the protocol) taken on a fluorescent dissecting microscope. Lateral views of stage 13 (A) and stage 15 (B) *srp-Gal4,uas-GFP;crq-Gal4,uas-GFP* embryos. Lateral view of a stage 15 *srp-Gal4,uas-GFP/+;crq-Gal4,uas-GFP/uas-N17Rac* embryo (C) in which hemocytes have failed to migrate out of the head, demonstrating what embryos look like when hemocytes are not apparent along their migratory routes. Lateral view of a stage 17 *srp-Gal4,uas-GFP;crq-Gal4,uas-GFP* embryo showing the convoluted structure of the gut at this stage of development (D); the onset of muscle contraction prevents live imaging of embryos beyond this stage of development. Ventral views of stage 13 (E) and stage 14 (F) *srp-Gal4,uas-red stinger* embryos showing dispersal of hemocytes with fluorescently labeled nuclei. Observation of hemocytes by fluorescence at stage 2.11 of the protocol is a prerequisite to obtain excellent images; anterior is to the right for all images.


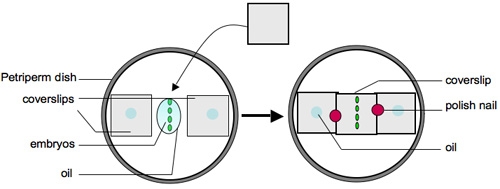
 **Figure 3. Mounting of embryos on a Petriperm/Lumox dish**.Two 18x18mm coverslips (thickness 1) are stuck to the bottom face of the Petriperm dish using a small drop of oil, separated by about 1cm as shown. Embryos are then lined up ventral side up with their long (anterior-posterior) axis parallel to the edges of the coverslips and covered with a small drop of oil. Once the oil has spread to fill the gap between the two coverslips a third coverslip (18x18mm thickness 1) is gently placed on top of the oil-covered embryos using the two previously adhered coverslips as a bridge to prevent the embryos from being squashed. This coverslip is then glued to the two coverslip bridges using two small drops of nail polish. Once set, the embryos can be imaged on an upright or inverted microscope with the objective lens focusing down through the coverslip (as opposed to through the Petriperm membrane).


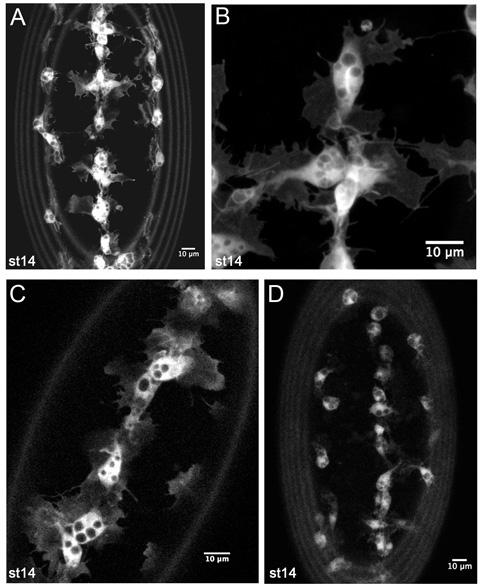
 **Figure 4. Representative results from live imaging of GFP labeled hemocytes**.Z-projections of hemocytes on the ventral side of a stage 14 *srp-Gal4,uas-GFP;crq-Gal4,uas-GFP* embryo (A-B). (A) is a lower magnification image such as used to monitor hemocyte developmental migrations in timelapse movies. (B) is a higher magnification still of hemocytes on the ventral midline, showing fine details of their morphology. (C) is a single 1 m slice of hemocytes on the ventral midline in a stage 14 *srp-Gal4,uas-GFP/+;crq-Gal4,uas-GFP/+* embryo, revealing that lower copy numbers of Gal4 drivers and uas constructs are also sufficient to generate good images. (D) displays a z-projection of hemocytes in a stage 14 *crq-Gal4,uas-GFP* embryo. Here hemocyte protrusions are less obvious due to lower expression of GFP but it is still possible to make movies and track hemocyte migration with this combination of Gal4 driver and uas construct. Images were taken on a Leica LSM510 confocal microscope; anterior is up in all images; the rings at the periphery of images are caused by vitelline membrane autofluorescence.


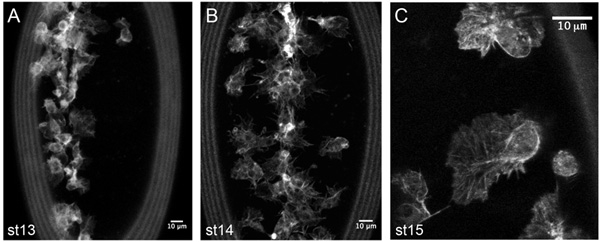
 **Figure 5. Representative results from live imaging of GMA expressing hemocytes**.Z-projections of hemocytes on the ventral midline of stage 13 (A) and stage 14 (B) *srp-Gal4,uas-GMA* embryos, taken from timelapse movies to show developmental migrations of hemocytes. Detailed information on actin dynamics can be obtained by higher magnification imaging of GMA expressing hemocytes (C). GMA consists of GFP fused to the actin-binding domain of moesin and labels actin filaments. Anterior is up in all images; images were taken on a confocal microscope.

## Discussion

The most important elements of this procedure are the selection of healthy embryos with clearly labeled hemocytes and to mount them carefully without damaging them. Once the embryos are in the halocarbon oil they are resistant to dehydration and once mounted can be imaged for several hours. In our hands we can image hemocytes for three hours, with negligible dehydration of the embryo or obvious photo-damage, taking a z-stack of images every three minutes on our Zeiss LSM510 confocal microscope with a 40X objective. Since hemocytes are highly dynamic there may a trade-off between spatial and temporal resolution: if a very high-resolution image is needed the hemocyte and structures within it may move during the scan. The precise details of how images are subsequently collected will be determined by the experimental questions to be addressed.

We typically use *uas-GFP* or *uas-GMA*^3^ to label hemocytes; *uas-GMA* is particularly useful since, in addition to marking hemocytes, it provides a read-out of actin filament dynamics. Other uas constructs may be used to probe hemocyte behavior: for example uas-cherry constructs^9^ can be exploited as alternative fluorophores or *uas-tau-GFP*^10^ could be used to visualize the microtubules within a hemocyte. Uas constructs with a nuclear label may be particularly valuable for automated tracking of hemocyte movements (Figure 2E-F). Two-color imaging is also possible (e.g. one can label both the actin and microtubule cytoskeletons using *uas-cherry-moesin* and *uas-tau-GFP*, respectively, under the control of a hemocyte-specific Gal4 driver). As previously mentioned, a key determinant in live imaging of hemocytes is the Gal4 driver employed: as regards to current hemocyte-specific promoters *srp-Gal4*^2^ > *crq-Gal4*^11^ > *pxn-Gal4*^4^, with only *srp-Gal4* sufficient to label hemocytes when heterozygous, whereas at least two copies of *crq-Gal4* or *pxn-Gal4* are needed to observe hemocytes live. Nonetheless optimal imaging with *srp-Gal4* requires homozygosity (or the presence of an alternative Gal4 driver).

By following hemocytes live in this way it is possible to study their developmental dispersal from the head^8^ and observe how they interact with the cues that pattern this process, deposit matrix and engulf apoptotic corpses. Hemocytes also represent an interesting system with which to probe the machinery of cell migration; by examining the migration of hemocytes in embryos that lack components of the actin or microtubule cytoskeletons it is possible to understand their *in vivo* function more clearly (e.g. Rho GTPases^4, 12^). Exploitation of uas constructs to label these cytoskeletons provides yet more detailed information on their regulation.

We have modified this technique to probe hemocyte behavior in a variety of contexts such as their responses to laser-induced wounds4 and injection of fluorescently labeled bacteria^13^. Furthermore this method can easily be adapted to image other cell types by choosing different Gal4 drivers. For example we have previously imaged dorsal closure using epithelial Gal4 drivers^14, 15^. The application of this technique to other cell types is limited by the strength of the Gal4 driver, the position of the cells to be imaged within the embryo and the type of microscope used for imaging; multiphoton confocal microscopes enable the user to image cell behavior deep within the embryo (e.g. germ cell transepithelial migration^16^), whereas conventional confocal microscopes are unable to reach these depths (this is why we image along the ventral midline where hemocytes are very superficial, trapped between the developing ventral nerve cord and epidermis).

The strength of this protocol is that it provides an environment to maintain embryos in a healthy condition for live imaging. While a degree of manual dexterity is required to position the embryos, it is an easy technique to master and quickly gives reproducible results and can easily be modified using different Gal4 drivers and uas constructs to probe many different aspects of hemocyte biology. Additionally, the protocol is not restricted to hemocytes since the use of alternative Gal4 drivers enables the behavior of other tissue types to be analyzed.
